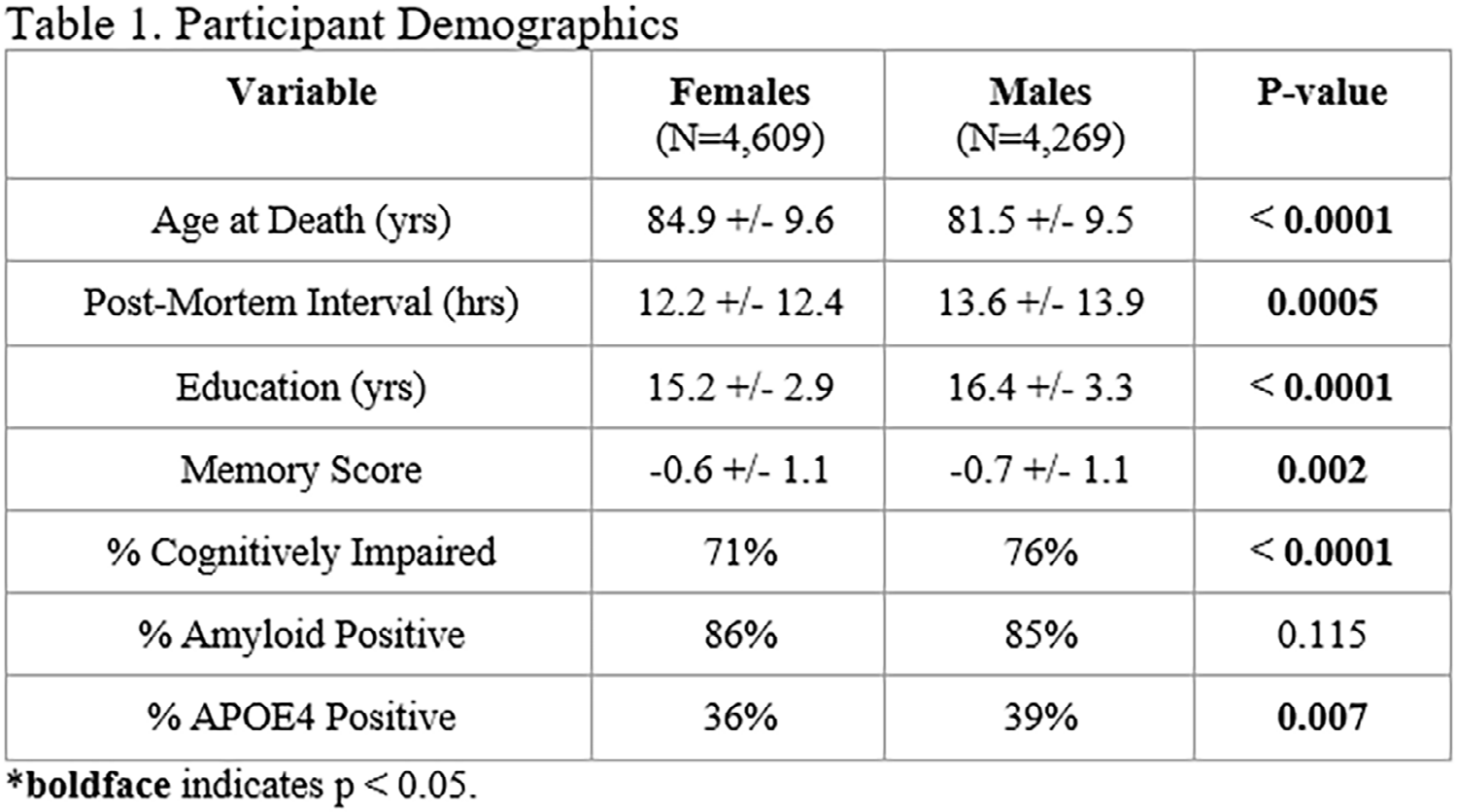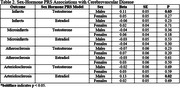# Genetically predicted levels of sex hormones are associated with cerebrovascular disease pathology in the Alzheimer’s disease brain

**DOI:** 10.1002/alz.092453

**Published:** 2025-01-03

**Authors:** Julia B. Libby, Gary Beecham, Thomas J. Montine, Arthur W. Toga, Michael L. Cuccaro, C Dirk Keene, Eric B Larson, Paul K. Crane, Shubhabrata Mukherjee, Matthew S. Panizzon, Sarah A Biber, Walter A. Kukull, David A. Bennett, Julie A. Schneider, Lisa L. Barnes, Philip L. De Jager, Vilas Menon, Timothy J. Hohman, Logan C. Dumitrescu

**Affiliations:** ^1^ Vanderbilt Memory & Alzheimer’s Center, Vanderbilt University Medical Center, Nashville, TN USA; ^2^ Dr. John T. Macdonald Foundation Department of Human Genetics, University of Miami Miller School of Medicine, Miami, FL USA; ^3^ Department of Pathology, Stanford University School of Medicine, Stanford, CA USA; ^4^ Laboratory of Neuro Imaging, Stevens Neuroimaging and Informatics Institute, Keck School of Medicine, University of Southern California, Los Angeles, CA USA; ^5^ The John P. Hussman Institute for Human Genomics, University of Miami, Miami, FL USA; ^6^ University of Miami Miller School of Medicine, Miami, FL USA; ^7^ Department of Laboratory Medicine and Pathology, University of Washington, Seattle, WA USA; ^8^ Department of Medicine, University of Washington, Seattle, WA USA; ^9^ Department of Medicine, University of Washington School of Medicine, Seattle, WA USA; ^10^ University of California, San Diego, La Jolla, CA USA; ^11^ Center for Behavior Genetics of Aging, University of California, San Diego, La Jolla, CA USA; ^12^ National Alzheimer’s Coordinating Center, University of Washington, Seattle, WA USA; ^13^ Rush University, Chicago, IL USA; ^14^ Rush Alzheimer’s Disease Center, Rush University Medical Center, Chicago, IL USA; ^15^ Rush University Medical Center, Chicago, IL USA; ^16^ Cell Circuits Program, Broad Institute, Cambridge, MA USA; ^17^ Department of Neurology, Center for Translational and Computational Neuroimmunology, Columbia University Medical Center, New York, NY USA; ^18^ Broad Institute of MIT and Harvard, Cambridge, NY USA; ^19^ Center for Translational & Computational Neuroimmunology, Department of Neurology, Columbia University Irving Medical Center, New York, NY USA; ^20^ Vanderbilt Genetics Institute, Vanderbilt University Medical Center, Nashville, TN USA; ^21^ Department of Neurology, Vanderbilt University Medical Center, Nashville, TN USA; ^22^ Vanderbilt Memory and Alzheimer’s Center, Vanderbilt University Medical Center, Nashville, TN USA

## Abstract

**Background:**

Cerebrovascular pathology frequently co‐occurs with Alzheimer’s disease (AD) pathology and the combinations of these forms of pathology may underly AD dementia. Sex hormones influence many aspects of cerebrovascular systems and may contribute to cerebrovascular pathology, but many studies of aging and AD do not measure hormones. Therefore, in this study, we explored whether a polygenic score predicting sex hormone levels relates to cerebrovascular pathology in the AD brain.

**Method:**

Sex‐specific Polygenic Risk Scores (PRS) of the sex hormones testosterone (N_males_: 146,339; N_females_: 142,778) and estradiol _(_N_males:_ 147,690; N_females:_ 163,985) were built using a linkage‐disequilibrium (LD) clumping method from published sex‐stratified GWAS from UK Biobank, using individuals with an age range of 40‐69 years old. Each PRS was scaled before analysis. Genetic data and harmonized cerebrovascular pathology data were leveraged from non‐Hispanic White autopsy participants from three independent studies of aging and AD: Adult Changes in Thought (ACT), the National Alzheimer’s Coordinating Center (NACC), and the Religious Orders Study/Rush Memory and Aging Project (ROSMAP) (N_males_: 4,269; N_females_: 4,609). Characteristics of the participants are described in Table 1. Sex‐specific logistic regression models were used to evaluate whether each PRS relate to cerebrovascular disease (CVD) outcomes, including presence or absence of macroscopic infarcts, microinfarcts, atherosclerosis, or arteriolosclerosis. Covariates included age at death, last clinical diagnosis before death, and education.

**Result:**

Higher genetically predicted levels of testosterone in males were associated with the presence of macroscopic infarcts (Table 2; b = 0.11, p = 0.03). Interestingly, higher genetically predicted levels of estradiol in males were associated with the presence of arteriolosclerosis (Table 2; b = 0.13, p = 0.02). No significant associations were observed for the other CVD outcomes in men and none in women. Significant results did not survive corrections for multiple comparisons.

**Conclusion:**

The results show that a PRS of sex hormones can be used to explore the effect of sex hormone levels on cerebrovascular pathology as it pertains to AD. The results also support the need for more studies investigating the role of sex hormones on sex‐specific phenotypes of cerebrovascular pathology in the presence of AD. Future work is needed to better understand the mechanisms behind the observed associations.